# Supplementary material for: Clinical characteristics of COVID-19 patients hospitalized at Clinique Ngaliema, a public hospital in Kinshasa, in the Democratic Republic of Congo: A retrospective cohort study
Source: PLoS One. 2020 Dec 18;15(12):e0244272. doi: 10.1371/journal.pone.0244272 (PMC7748279; doi:10.1371/journal.pone.0244272)
Supplement: S1 Table — (DOCX) [file pone.0244272.s001.docx]

| **S1 Table. Factors associated with antimalarial use in COVID-19 patients** | | | | | |
| --- | --- | --- | --- | --- | --- |
| **Variables** | | **Univariate OR** | **p value** | **Multivariate OR** | ***P* value** |
| Age_years | | 1.02 (0.98- 1.04) | 0.228 | - | - |
| Sex | Male | 1.12 (0.45- 2.8) | 0.808 | - | - |
|  | Female | 1 |  |  |  |
| Presence of Comorbidity | Yes | 1.06 (0.42- 2.67) | 0.906 | - | - |
|  | No | 1 |  |  |  |
| SpO2_%, median (IQR) | | 0.89 (0.79- 1.01) | 0.077 | - | - |
| Fever | Yes | 8.68 (3.44- 21.93) | <0.0001* | 4.04 (1.42- 11.51) | 0.008* |
|  | No | 1 |  |  |  |
| Cough | Yes | 2.3 (0.84- 6.34) | 0.106 | - | - |
|  | No | 1 |  |  |  |
| qSOFA | | 2.29 (1.24- 4.24) | 0.008* | 1.67 (0.82- 3.41) | 0.157 |
| SIRS | Yes | 1.26 (0.45- 3.54) | 0.655 | - | - |
|  | No | 1 |  |  |  |
| Shortness of breath | Yes | 1.91 (0.73- 4.95) | 0.188 | - | - |
|  | No | 1 |  |  |  |
| Therapeutic itinerary | Transferred from non CTC | 3.78 (1.24- 6.54) | 0.005* | 3.12 (1.18- 8.16) | 0.021* |
|  | Attended directly Cl.Ng. | 1 |  |  |  |
| History of exposure to a confirmed or suspected COVID-19 case | Clear history of exposure | 0.21 (0.09- 0.49) | 0.0003* | 0.34 (0.13- 0.88) | 0.027* |
|  | Unknown exposure | 1 |  |  |  |
| SpO2: peripheral oxygen saturation, qSOFA= Quick Sequential Organ Failure Assessment, SIRS: Systemic Inflammatory Response Syndrome, OR: odds ratio | | | | | |
