# Supplementary material for: Clinical characteristics of COVID-19 patients hospitalized at Clinique Ngaliema, a public hospital in Kinshasa, in the Democratic Republic of Congo: A retrospective cohort study
Source: PLoS One. 2020 Dec 18;15(12):e0244272. doi: 10.1371/journal.pone.0244272 (PMC7748279; doi:10.1371/journal.pone.0244272)
Supplement: Attachment — Submitted filename: Response to Reviewers_3.docx [file pone.0244272.s002.docx]

06^th^ December, 2020

Dear Editor,

Thank you for your interest towards our paper entitled “**Clinical characteristics of COVID-19 patients hospitalized at Clinique Ngaliema, a Public hospital in Kinshasa, in the Democratic Republic of Congo: a retrospective cohort study**.” (manuscript ID: PONE-D-20-28772).

Kindly see below our response to the point raised:

1. Based on the information you've provided, we've drafted for your approval the following proposal to update your Data Availability Statement:

"The data underlying this study cannot be shared publicly, as it is the property of the Clinique Ngaliema Hospital. For these reason, the hospital would prefer to share data for research purposes, upon reasonable request, accompanied by a research proposal with a detailed description of the objectives of the study, sent directly to the steering committee of Clinique Ngaliema Hospital at the following e-mail address: [infor@cliniquengaliema.org.](mailto:infor@cliniquengaliema.org.) The authors did not receive any special privileges in accessing the data."

Please confirm whether the above proposal is accurate, and if so, whether we may update your statement on your behalf.

R/ I hereby confirm that this sentence is accurate. Please, feel free to update my statement on my behalf.

Kind regards,

Junior Matangila Rika, PhD

Department of Tropical Medicine, Kinshasa University

E-mail: [matangilaj@yahoo.fr](mailto:matangilaj@yahoo.fr)

Mobile: +243-970756890
